# A Comparison of Independent Event-Related Desynchronization Responses in Motor-Related Brain Areas to Movement Execution, Movement Imagery, and Movement Observation

**DOI:** 10.1371/journal.pone.0162546

**Published:** 2016-09-16

**Authors:** Jeng-Ren Duann, Jin-Chern Chiou

**Affiliations:** 1 Institute of Cognitive Neuroscience, National Central University, Zhongli, Taoyuan District, Taiwan; 2 Institute for Neural Computation, University of California San Diego, La Jolla, California, United States of America; 3 Department of Electrical Engineering, National Chiao Tung University, Hsinchu, Taiwan; University of Electronic Science and Technology of China, CHINA

## Abstract

Electroencephalographic (EEG) event-related desynchronization (ERD) induced by movement imagery or by observing biological movements performed by someone else has recently been used extensively for brain-computer interface-based applications, such as applications used in stroke rehabilitation training and motor skill learning. However, the ERD responses induced by the movement imagery and observation might not be as reliable as the ERD responses induced by movement execution. Given that studies on the reliability of the EEG ERD responses induced by these activities are still lacking, here we conducted an EEG experiment with movement imagery, movement observation, and movement execution, performed multiple times each in a pseudorandomized order in the same experimental runs. Then, independent component analysis (ICA) was applied to the EEG data to find the common motor-related EEG source activity shared by the three motor tasks. Finally, conditional EEG ERD responses associated with the three movement conditions were computed and compared. Among the three motor conditions, the EEG ERD responses induced by motor execution revealed the alpha power suppression with highest strengths and longest durations. The ERD responses of the movement imagery and movement observation only partially resembled the ERD pattern of the movement execution condition, with slightly better detectability for the ERD responses associated with the movement imagery and faster ERD responses for movement observation. This may indicate different levels of involvement in the same motor-related brain circuits during different movement conditions. In addition, because the resulting conditional EEG ERD responses from the ICA preprocessing came with minimal contamination from the non-related and/or artifactual noisy components, this result can play a role of the reference for devising a brain-computer interface using the EEG ERD features of movement imagery or observation.

## Introduction

Movement imagery (MI), which is the mental simulation of a given motor action, elicits the same responses in common neural substrates as does the actually executed action [1]. Ample evidence has shown that kinesthetic MI activates the same neural networks as motor planning and thus relies on the same motor structures as movement execution [2, 3]. As a result, mental practice through MI has been widely applied in sports training and rehabilitation, especially rehabilitation for stroke patients [4–8]. In neurorehabilitation, some neuroimaging-based markers are used to detect the brain activity associated with the MI performance so as to facilitate the performance of stroke rehabilitation training protocols or devices, or simply to provide feedback in visual or auditory forms.

Among the neuroimaging-based markers most applicable for use in the online monitoring of MI task performance are electroencephalographic (EEG) event-related desynchronization (ERD) patterns. In general, MI task performance induces an oscillatory EEG process of desynchronization (also known as mu suppression or sensorimotor rhythm, SMR) in the EEG alpha (8–12 Hz) and beta (16–30 Hz) frequency bands, time-locked to the event onset, similar to that induced by the actual motor execution (ME) [9–11]. Such EEG ERD activity has also been found in the process of observing biological movements performed by someone else, thus movement observation (MO) [12–17]. In line with these findings, the detection of EEG ERD responses induced by MI as well as by MO task performance have been incorporated into brain-computer interface- (BCI-) controlled neurorehabilitation apparatuses or protocols for stroke rehabilitation training [3, 7, 18, 19].

Although MI and MO have been used for stroke rehabilitation training based on the fact that they induce EEG ERD responses in the alpha and beta frequency bands of motor-related neural substrates similar to those elicited by true ME, how reliable the EEG ERD responses induced by the MI and MO are in comparison to those elicited by ME has yet to be sufficiently studied [19]. For example, how robust the ERD induced by different motor condition, how long after the motor cue the ERD response may start, and how deep the power suppression can be are all indispensable for devising a BCI application that utilizes these movement conditions. Therefore, the goal of the present study was to use an EEG experiment to compare the EEG ERD responses in motor-related brain areas induced by the different movement conditions.

In addition to conducting an EEG experiment with multiple movement conditions, we also used independent component analysis (ICA) to identify the common neural substrates activated by the performance of the three movement conditions. In the past, ICA has been used to determine the EEG activities that occur in a given neural substrates when different tasks are performed. In principle, ICA can linearly separate independent neural activities from muscle and eye artifacts, even when those activities and artifacts are acquired together in the form of multi-channel EEG data [20]. Such ICA decomposition reveals both the temporal activity and corresponding specific topography of the independent neural activity, indicating its spatial patterns on the scalp and roughly reflecting where in the brain the activity is occurring in temporal terms. Furthermore, if the temporal activity includes the brain EEG responses to more than one condition, it is possible to compare the evoked potentials or other EEG derivatives associated with the different conditions in the same EEG topography or, roughly, the same neural circuits. For example, Chou and colleagues compared the EEG power changes, phase-locking values, event-related coherences, and so forth evoked in the frontal midline, central parietal, and occipital brain areas of single subjects associated with different phases of the working memory process (i.e., visual processing, encoding, and retrieval) [21].

On the other hand, ICA can also be used to determine the brain activities of common brain circuits across different subjects or subject groups. Onton and colleagues compared the event-related potentials (ERPs) associated with left and right motor-related activity in young and old normal subjects [22]. They first decomposed single-subject EEG data using ICA to extract independent neural activities. Next, they clustered the equivalent independent components across different subjects based on the scalp map, ERP, and time-frequency features of each of the independent EEG components. Finally, they were able to derive the group ERPs for comparison by averaging the ERPs of the clustered equivalent components across the subjects within the same groups. Therefore, it is possible that ICA can identify not only the brain activities evoked in similar brain circuits within the same subjects but also those evoked across different conditions or tasks, or even across different subjects or subject groups for further comparison.

In this study, the same motor-related brain areas were identified by applying ICA to the EEG data collected during the three different movement conditions, which were performed consecutively in a pseudorandomized order in the same task runs. In so doing, we expected (1) to exclude contamination from other brain or non-brain activities that were irrelevant to the sensorimotor rhythms induced by the performance of the three motor conditions, and (2) to collect sufficient data to make a direct comparison between the EEG ERD responses in shared motor-related cortices induced by the three different motor conditions. The results of this study may thus make it possible to determine the best scenarios for eliciting a given EEG ERD responses induced by MI and MO in comparison to those induced by ME with minimum artifact contamination. The resultant EEG ERD patterns could also be informative for designating the parameters, such as the onset time, duration, and depth of suppression of the ERDs, for devising a BCI-controlled stroke rehabilitation device based on the MI or MO performance.

## Methods

### Subjects

Thirteen normal healthy right-handed subjects (five females, mean age 24 ± 3 years old) participated in this study. The handedness was determined according to the Edinburgh inventory (www.brainmapping.org/shared/Edinburgh.php). All the subjects were recruited from the campus of National Chiao Tung University, Hsinchu, Taiwan. They all had no history of neurological diseases or central or peripheral nervous system injury prior to the experiment and were all naive to any form of BCI neurofeedback. All the subjects provided signed written consent before the experiment, and they were all paid for their participation. The Institutional Review Board (IRB) of China Medical University Hospital (http://www.cmuh.cmu.edu.tw) in Taichung, Taiwan, approved the experimental protocol (IRB no. DMR100-IRB-221). The experiment was conducted according to the principles expressed in the Declaration of Helsinki.

### Experimental paradigm

**Fig 1:**
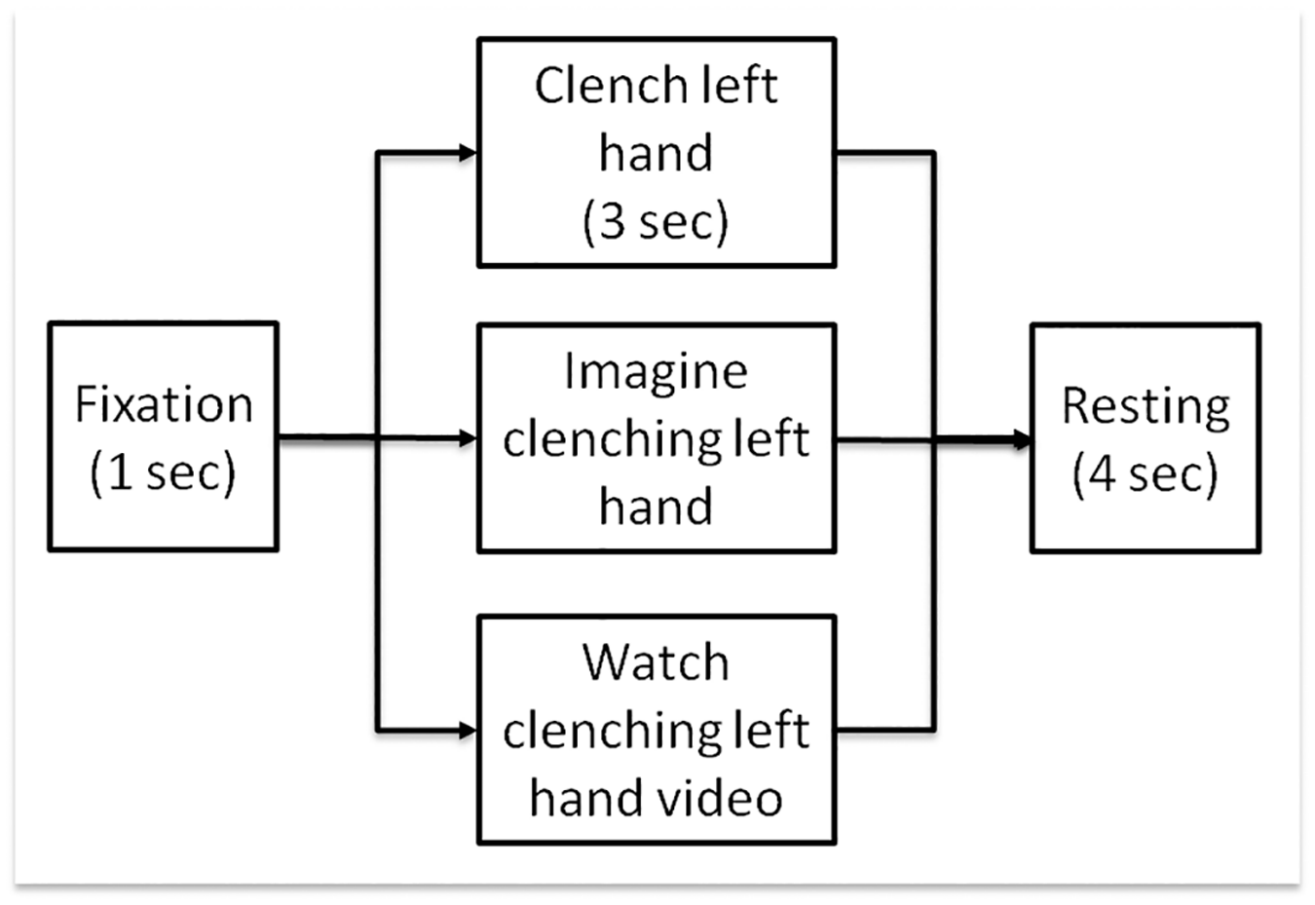
Experimental protocol of the multitask EEG experiment with three different motor conditions, namely motor execution (ME), motor imagery (MI), and movement observation (MO). Each trial of experiment consisted of 1-sec fixation, 3-sec task performance and 3–5 sec (4-sec on average) resting periods. During the 3-sec task performance, the participants were requested to either physically clench their left hand once, imagine clenching their left hand once, or watch a video clip of a human left hand clenching once, during the entire 3-sec duration.

In order to compare the EEG ERD responses induced by different motor tasks, an EEG experiment consisting of three different movement conditions, namely, movement execution (ME), movement imagery (MI), and movement observation (MO), was conducted. Each EEG experimental run consisted of 15 trials, with five trials for each of the three movement conditions. The three conditions were presented in a pseudoransomized order. Each trial started with a 1-sec fixation window, during which a white cross was displayed at the center of a computer screen for the given subject to fixate on. Next, a visual cue was displayed indicating which of the three motor activities should be performed, and the subject was asked to perform the given action accordingly (Fig 1). For the ME condition, a text message stating “Clench Left Hand” was displayed and maintained on the computer screen for 3 sec, and the subject was requested to slowly make a fist once with his/her left (non-dominant) hand during the entire 3-sec window. For the MI condition, a text message stating “Imagine clenching left hand” was displayed, and the subject was asked to kinesthetically imagine clenching his/her left hand once during the entire 3-sec window. During the MO trials, a 3-sec video clip from a first-person perspective showing a real human left hand slowly clenching once was played to the subject, and the subject was asked to watch the video carefully without making any physical movement to reproduce the action. The video clip revealed only the left hand of the actor; no other body parts were visible. Finally, a 3- to 5-sec (4-sec on average) resting period was appended to make an average 8-sec time window for each single trial. As a result, each EEG run lasted for 120 seconds. Each subject completed four EEG runs, resulting in 60 trials, with 20 trials for each motor condition. In order to make sure that the subjects did not move their hands during the MI and MO trials, the subjects were asked to practice not moving them when they were seated for EEG capping, and their hands were visually observed by an experimenter to prevent any movements during the MI and MO trials. The experiment was started only when the subjects completely conformed to the requirements of the task.

### EEG acquisition

The EEG data were acquired using a Neuroscan SymAmp2 (Computmedics Ltd., Victoria, Australia) with 64 channels, including two EOG channels, via unipolar montage following the International 10–20 system. All electrodes were referenced to the linked mastoids, and the ground channel was placed at AFz. Electrode impedance was maintained below 5K Ohm and checked before and after the recordings. The EEG was recorded with a band-pass filter of 0.1–250 Hz and digitized at the sampling rate of 1000 Hz.

### Data preprocessing

The acquired EEG signals for each individual subject were first examined by visual inspection to remove bad EEG channels as well as bad EEG portions with amplitudes greater than 100 uV. Then, the EEG data were filtered using a band-pass filter of 1–50 Hz and downsampled to 250 Hz. After preprocessing, the EEG data of all four runs were concatenated for further analysis.

### ICA decomposition

Before applying independent component analysis (ICA) to the data, the two EOG channels were removed. The concatenated preprocessed data consisting of the EEG data of all 60 motor trials mixed together were then subjected to ICA decomposition. This was done to determine which independent motor-related EEG components were common to all three different motor conditions. As a result, we were able to compare the EEG ERD responses in the same neural substrates underlying the performance of the three different motor tasks. The ICA decomposition was conducted using an infomax ICA algorithm as implemented in EEGLAB (http://sccn.ucsd.edu/EEGLAB) [23]. It was conducted in order to separate the independent brain EEG processes from those of artifactual components, such as eye artifacts (blinking and lateral eye movement), muscle activity, environmental noise, etc. In general, the number of components can be decomposed using ICA is equal to or less than the EEG channel number. Thus, the EEG data of 12 out of 13 subjects were decomposed into 62 independent components (full-rank decomposition), and the remaining subject’s data were decomposed into 61 independent components because one EEG channel had been removed before ICA decomposition due to excessive contamination.

### Clustering independent components

In order to find the motor-related independent components from all the subjects and compute group ERDs associated with different motor conditions, all the independent components obtained using ICA decomposition were further subjected to a source localization process using the DIPFIT2 plug-in in the EEGLAB toolbox [24]. The independent components with residual variance in the source localization process exceeding 15% were removed from the further analysis. A total of 525 independent components out of the original 805 were removed via this process. The removed components included those with single-channel activation topography or an EEG topography that could not be accounted for by single dipoles [25].

The remaining 280 independent components from all 13 subjects were then analyzed using the EEGLAB study analysis function for clustering the equivalent independent EEG components from different individual subjects. The clustering process needed to first compute the feature variables of the (1) component topography, (2) event-related time-frequency plot (defined as event-related spectral perturbation (ERSP) in EEGLAB), and (3) (x, y, z) coordinates of the dipole location. The feature variables of the component topography and time-frequency plot were then further summarized using the first 10 principal components. Finally, a K-mean clustering algorithm was used to categorize the independent components into 15 clusters based on the 23-dimension feature space, with one additional outlier cluster for those independent components that failed to be clustered into any of the 15 resultant clusters. However, it was possible that some subjects might contribute more than one component into the target component cluster. When more than one component was found in a given subject, we further computed the time-frequency plots of the components from this subject in the cluster and manually selected the one most resembling the mu-rhythm ERD. The purpose of doing so was to ensure that each subject only contributed one component to the target component cluster.

### Determining main frequency band of motor ERD

Within the 15 component clusters, the one with its topography mainly covering the right motor areas, which are activated by movement of the left-hand, was selected. Then, the time-domain signals of the selected components were segmented into 4-sec epochs extending from 1 sec before to 3 sec after the onset of the given visual cue. The mean component time-frequency plot was computed using all epochs (including trials from all three motor conditions) from all the selected components of the 13 subjects. The main purpose of computing this grand mean time-frequency plot was to determine the main frequency band with the most pronounced ERD in the EEG data.

### Comparing conditional ERDs

After the target component cluster was determined and the one equivalent independent component of right motor-related cortex from each subject was identified, the epochs were further separated into three types according to the different motor conditions (ME, MI, and MO) in order to compute the group mean ERD of each motor condition. The conditional ERD was computed across all trials within the same condition and across all subjects. We further computed the significance of power suppression across various time points for the selected frequency band relative to the baseline for different motor conditions. In addition, the time-point-by-time-point differences in EEG power suppression for the different motor conditions were further compared using paired t-tests to further test if the EEG power suppression caused by the MI and MO conditions was as reliable as the power suppression caused by real motor execution.

### Computing arrival time of motor ERD

In order to evaluate whether the EEG power suppression caused by the MI and MO conditions happened at the same time as the power suppression caused by motor execution, the arrival time of the EEG power suppression for each of the conditions was computed. The arrival time was defined as the time at which the power reached the half maximum of the overall suppression level from the baseline. The arrival time was derived first at the single-subject level by averaging across the trials for a given motor condition and then averaging across the entire subject population. The means and standard deviations of the conditional arrival times were obtained and compared across different motor conditions using a two-sample t-test.

## Results

### ICA decomposition and clustering

**Fig 2:**
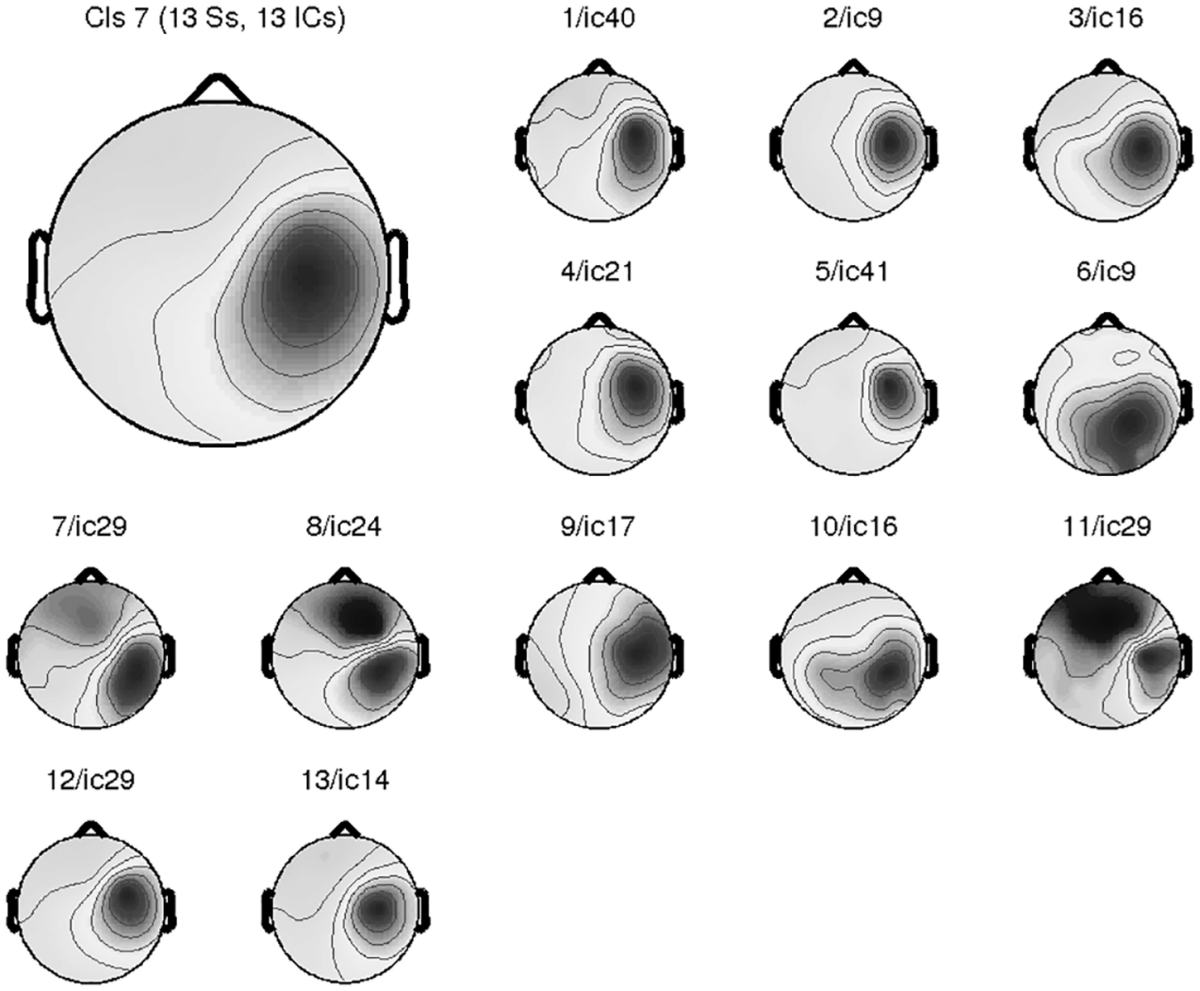
Average topography of the selected components from all participants (one for each). The upper left gives the average topography of all 13 selected components; others show the topography of right motor-related EEG component for each participant.

The preprocessed EEG data were decomposed into 62 independent components for all the subjects except for one, whose EEG data were decomposed into only 61 components due to the exclusion of one noisy channel prior to the ICA decomposition. After clustering analysis, one of the independent EEG components showing the EEG activity in the right motor-related cortex (i.e., the right mu component) was selected from each of the 13 subjects. Fig 2 shows the mean topography from the selected 13 component topographies (upper left corner) and the component topography of the selected component for each single subject (smaller plot in Fig 2).

### Grand mean time-frequency plot

**Fig 3:**
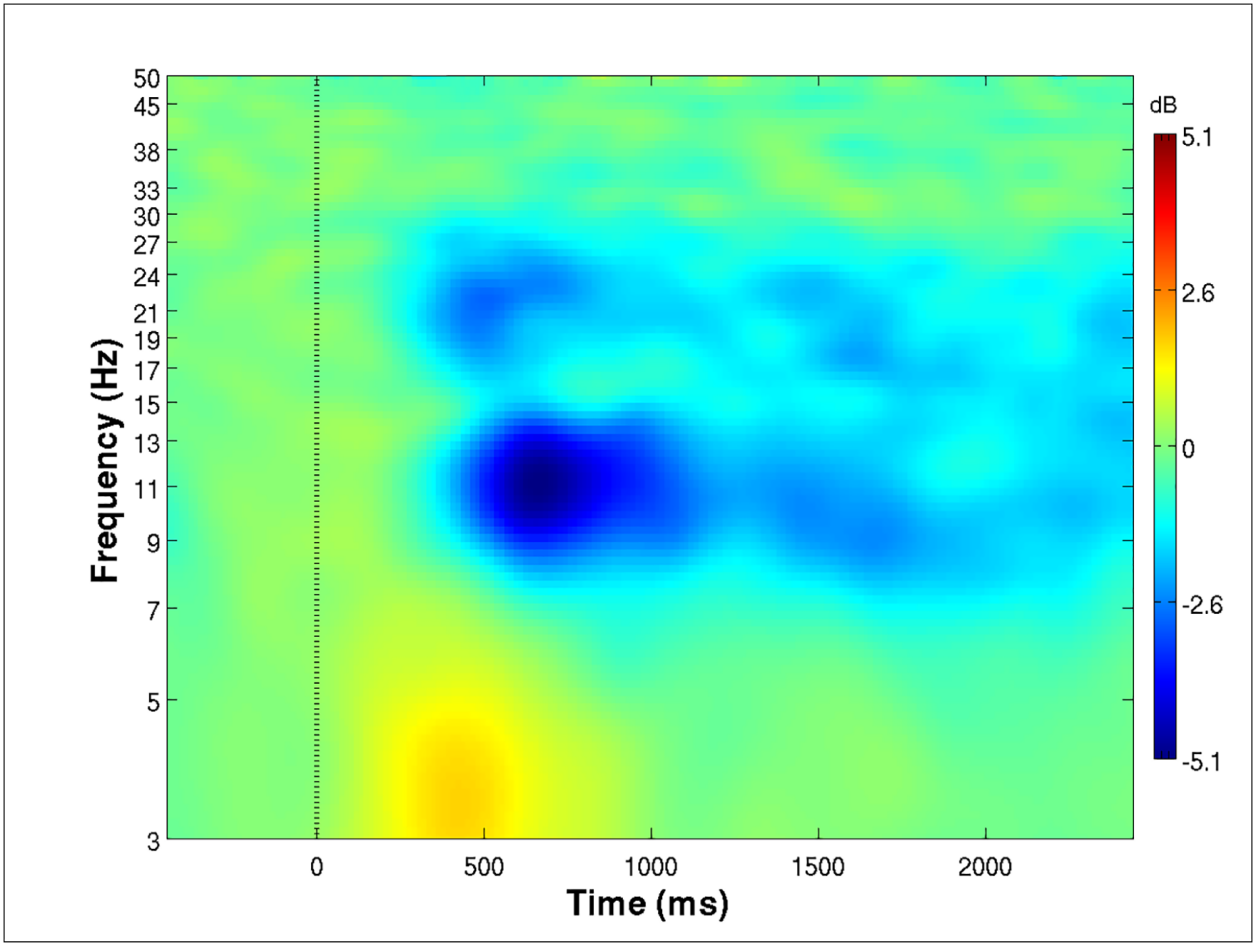
Mean time-frequency plot, time-aligned to the onset of execution cue (time zero), for all selected motor-related components. A mu-ERD pattern can be clearly seen in the plot, with the main suppression frequency band in the alpha (8–13 Hz) range. The suppression starts, on average, at 500 ms after the onset of execution cue.

In order to select the frequency band with the most pronounced ERD responses, a grand mean time-frequency plot was computed and plotted by averaging the time-frequency plots of all the trials for all three motor conditions for all the subjects (Fig 3). As a result, the alpha frequency band from 8 to 13 Hz was found to yield the most significant ERD responses during the 500 to 1000 ms window after the onset of the task execution cue (time zero). Although the results also showed another power suppression peak (the beta power suppression) at around 22 Hz, the alpha frequency band still exhibited much more pronounced power suppression. This finding was consistent with the findings of Neuper et al., in which consistent alpha peaks were reported in the grand average frequency responses to the motor imagery process at the C3 and C4 channel locations [11]. In contrast, the average beta activity across subjects was more widespread, indicating higher inter-subject variability. Therefore, we selected the alpha frequency band to compute the conditional ERD patterns for further comparison.

### Comparison of conditional ERDs

**Fig 4:**
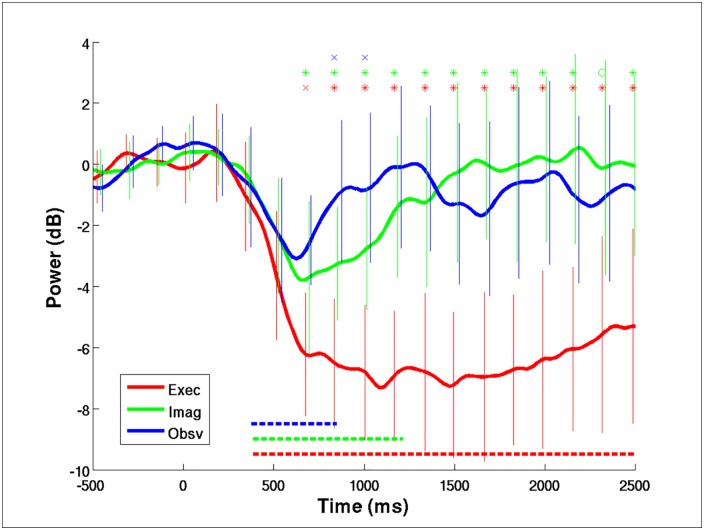
The grand mean conditional alpha ERD time courses, time-aligned to the onset of execution cue (time zero), across all subjects. The red line shows the mean alpha ERD time course of ME condition, the green shows the MI condition, and the blue shows the MO condition. The error bars show the standard deviation of the time course at each time point across all trials and subjects. The dotted lines at the bottom give the time windows with significant alpha suppression from the base line (at the significance level of 0.05), red for ME, green for MI, and blue for MO condition. The markers at the top of the plots indicate the significance level (x’s meaning p<0.05, o’s meaning p<0.01, and *’s meaning p<0.001) of the comparisons between conditional alpha ERD time courses at each time point. Red indicates the comparison between the conditions of ME and MI, green between ME and MO, and blue between MI and MO.

After the most pronounced frequency band of motor-related ERD response had been identified, the conditional ERD time courses were computed by averaging the ERD time courses from 8 to 13 Hz and across all subjects for each of the motor conditions. Fig 4 shows the mean conditional ERD derived from each of the three motor conditions: the resulting ERD time course for the ME condition is plotted in red; the time course for the MI condition is plotted in green; and the time course for the MO condition is plotted in blue. The error bars give the standard error of the mean ERD response across all trials for each motor condition. The dotted lines at the bottom (red for the ME condition, green for the MI condition, and blue for the MO condition) indicate the duration from the baseline for which the alpha power suppression was significant (at the significance level of p < 0.05). As shown in the figure, the ME condition, on average, resulted in reveals much longer alpha suppression as compared to other two motor conditions, with a duration lasting longer than 2000 ms. The MI condition caused a 600–700 ms time window of significant alpha power suppression, while the MO condition yielded only brief alpha suppression of less than 300 ms. In terms of the depth of alpha suppression, the strength of the ERD induced by the ME condition was almost double that of the ERDs induced by the MI and MO conditions. In the same time, the markers on top of the figure indicate the significance levels of the differences between the conditional ERD values at each time point, where x’s indicate p < 0.05, o’s p < 0.01, and *’s P < 0.001. The red markers refer to comparisons between the ME and MI conditions, the green markers refer to comparisons between the ME and MO conditions, and the blue markers refer to comparisons between the MI and MO conditions.

### Comparison of mean arrival times

**Fig 5:**
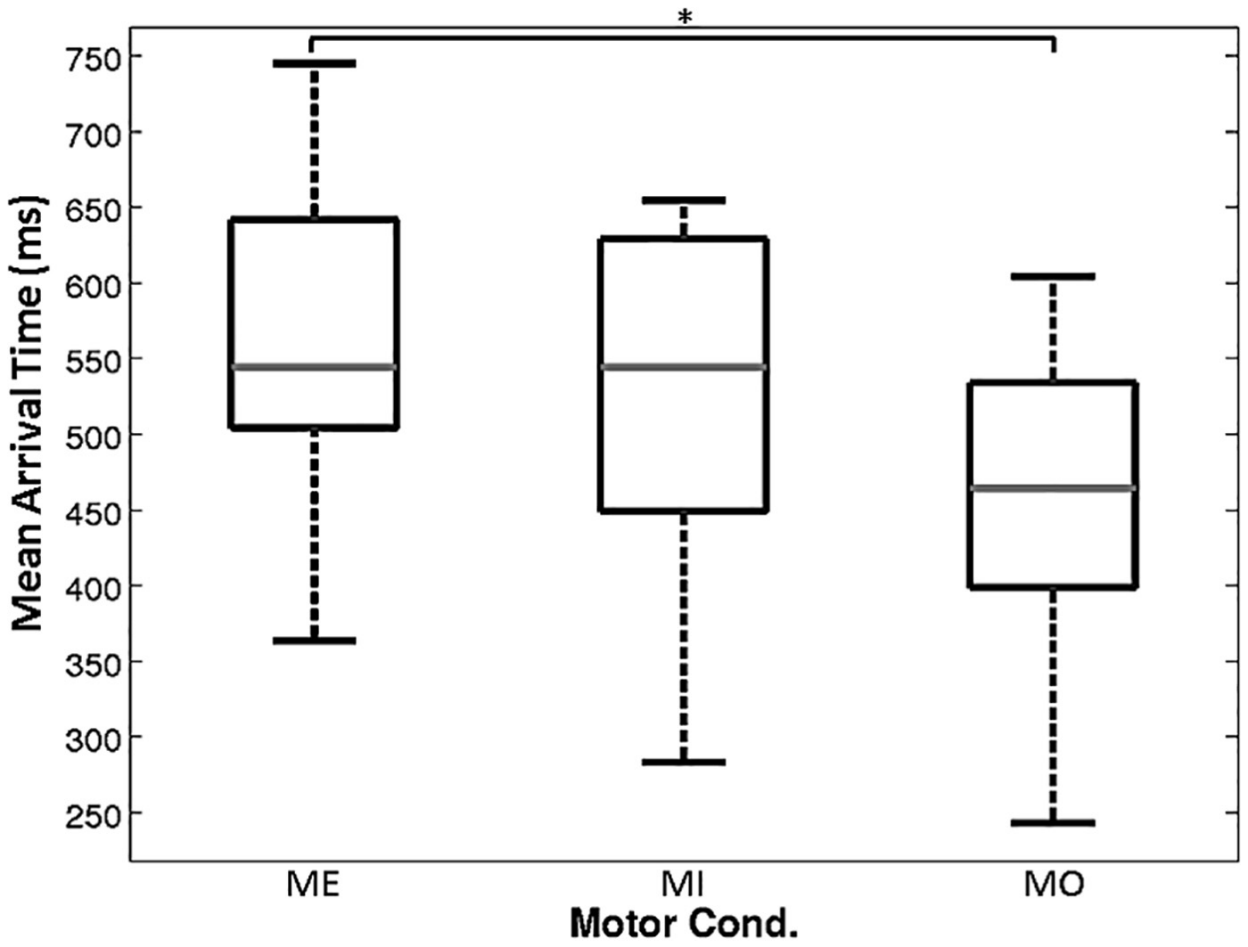
Mean arrival time of the alpha ERD time course of different motor conditions. The arrival time indicates when the alpha power reaches half the maximum of suppression. The results show that the arrival time of the MO condition is significantly faster than the arrival time of the ME condition (p<0.05).

The arrival time indicated the time at which the power of the alpha suppression reached the half maximum of the total alpha power suppression. Fig 5 shows the mean arrival times averaged across all trials for a given motor condition. In the results for comparisons between the different motor conditions, a significant difference can only be seen between the ME and MO conditions. Specifically, the arrival time for the MO condition is significantly faster than the arrival time for the ME condition (P<0.05).

## Discussion

To investigate how reliably EEG ERD responses can be induced by the performance of movement imagery and movement observation as compared to movement execution, we conducted a multitask EEG experiment involving all three different movement conditions with regard to the same action, clenching the left (non-dominant) hand. In the study of Leocani et al. [26], they revealed the contralateral dominance of ERD was more pronounced for the movement of dominant hand. As a result, performing the three movement conditions with non-dominant hand may create more confused ERD patterns to complicate the ERD detection in the EEG data. The three movement conditions were randomly arranged in an experiment run, such that the resulting EEG data contained the brain activities for the performance of all three different movement conditions. Next, ICA was used to decompose the concatenated EEG data for each individual subject to find the common independent EEG source activity associated with the three motor conditions. As shown in the results, the ME condition facilitated the most reliable, long-lasting EEG ERD activity in the alpha frequency band over the right motor-related cortex. In contrast, the ERD processes associated with MI and MO performances consisted only of brief responses with much shorter latencies and smaller suppression amplitudes. This is in line with a current study by Babiloni et al., showing that the electrocorticographic (ECoG) desynchronization was lower during movement observation than movement execution [27]. Nonetheless, the ERD responses for the MI task did show better detectability, with slightly longer and deeper power suppression from the baseline, than the ERD responses for the MO task. In the following discussion, we address the feasibility of comparing the ERD responses to different motor conditions derived from the common independent EEG sources in the motor-related cortex, as well as the possible causes for the different conditional ERD responses shown in our results.

### Common spatial distribution for different motor tasks

Determining the ERD responses for given brain regions facilitated by the performance of ME, MI, and MO tasks can be contentious because the spatial distribution of the brain activation maps associated with these three motor conditions remains controversial. For example, Wang et al. combined the methods of ICA and hierarchical clustering analysis (HCA) to find specific activated brain areas associated with visual processing, ME, and MI, as well as with the default mode network [28]. In their study, MI task performance activated the precuneus, the rostral part of dorsal lateral premotor area, and the rostral part of supplementary motor area (SMA), whereas ME task performance activated the bilateral primary motor area, the caudal part of dorsal lateral premotor area, the caudal part of SMA, the parietotemporal operculum, and the anteromedial part of the cerebellum. Hanakawa et al. revealed that the primary motor cortex, as well as the primary and secondary sensory cortices, was mainly activated during ME [29]. The premotor area, pre-SMA, and frontal eye field were found to be MI-dominant. In a most recent study by Babiloni et al., they also showed that the locations of reactive electrodes in an electrocorticographic recording, responding maximally to the ME and MO conditions, were highly overlapped [27].

However, most of the findings revealing spatial distinctions between the activation maps in response to MI and ME task performance are derived from fMRI or PET neuroimaging modalities with higher spatial resolution. Most EEG/MEG studies, in contrast, have found similar activation maps at the scalp level [14, 30]. More importantly, Miller et al. showed direct evidence of the involvement of the primary motor cortex in motor imagery task performance, however, to a smaller extent [31]. In addition, Mukamel et al. also showed overlapping activity in motor neurons and other cells of the medial frontal and temporal cortices in response to both executing and observing hand grasping actions [32]. On the other hand, some fMRI studies also showed similar activation maps in response to the performance of MI and ME [19]. Gerardin et al. also showed the primary motor cortex activation during MI but to a lesser extent (about 30% of the level observed during ME) [33]. Zich et al. also found similar group fMRI activation maps associated with MI and ME performance using both the left and right hands [34]. However, the group activation maps of the ME performance appeared to be more connected and solid, and larger in area under the same statistical threshold.

The “simulation theory” proposed by Jeannerod asserts that the covert and overt stage of an action should share similar neural circuits, specifically, those in which the action is simulated and executed [1]. Given the limited spatial resolution of the EEG neuroimaging modality used in this study and the aforementioned evidence, it is thus reasonable to explore the common independent EEG components, at a more blurred spatial scale, for the purpose of differentiating the EEG ERD responses in the alpha frequency band induced by the MI, ME, and MO motor tasks.

### Early ERD onset for MO condition?

According to a comparison of the arrival times of the ERD responses for the three motor conditions, the arrival time of the responses for the MO condition was significantly faster than that of the responses for the ME condition (Fig 5). The performance of an MO task has been shown to activate the mirror neurons in the human brain [35]. In addition, in a recent study, Mukamel et al. used single-cell recording technology to not only provide critical and direct electrophysiological evidence that humans have mirror neurons [32], but also to show that the human mirror neuron system extends beyond the ventral premotor cortex and inferior parietal lobule. Due to clinical considerations, they had to place electrodes in the brain areas of the cingulate cortex, SMA, pre-SMA, amygdala, hippocampus, parahippocampus gyrus, and entorhinal cortex, instead of monitoring the whole brain. Even with this limitation in placing electrodes, Mukamel and colleagues still confirmed the existence of mirror neurons in the brain areas of SMA, hippocampus, parahippocampal gyrus, and entorhinal cortex [32], as proposed in the work of Keysers and Gazzola [36]. Therefore, the performance of an MO task might activate the motor-related brain areas as well as the SMA because of the mirror neuron system. However, our results reproduced the findings of Gastaut and Bert [37] and Cochin et al. [13], in which the ERD recorded from the scalp locations C3 and C4 by observation of experimental hand grasp was reported to be reduced in amplitude and latency.

In a seminal work by Hanakawa et al. [29], the authors studied the fMRI time courses of the blood oxygenation level dependent (BOLD) responses to motor planning, motor imagery, and motor execution using a finger tapping task. According to the onsets of the peaks of fMRI time courses, they divided the brain activations into instruction-related and execution-related activities. The latter form of activity was mainly located in the primary motor cortex for actual movement, while the former form of activity was located in the SMA/pre-SMA, premotor cortex, anterior cingulate cortex, supramarginal gyrus, fusiform gyrus, etc., for motor planning and generation. Hanakawa and colleagues also showed that the MI task mainly activated the brain areas with their BOLD responses in line with the instruction-related activity and that the ME task activated the brain areas due to the execution-related activity [29].

Given the above evidence, we hypothesize that performances of MO, MI, and ME tasks facilitate, to different extents, different types of brain activity with instruction-related activity and execution-related activity located on opposite ends of a one-dimensional axis, where MO tasks cause activity located more toward the instruction-related side, ME tasks cause activity located more toward the execution-related side, and MI tasks cause activity located somewhere in between. Because MO tasks might more strongly activate the mirror neuron system and instruction-related brain activity, such tasks may be most effective at facilitating motor preparation activity but least effective at facilitating motor execution brain activity. As a result, among the three types of motor tasks in the present study, the ERD responses associated with the MO task had the fastest average response time but only very brief alpha power suppression because the common brain activation found in this study using ICA might better cover the execution-related side due mainly to the more pronounced brain electrophysiological activity associated with the motor execution condition [31]. An ME task, on the other hand, mainly activates brain activity in areas associated with motor execution, such as the primary motor cortex, dorsal premotor cortex, anterior parietal cortex, etc., which, according to Hanakawa et al. [29], had relatively late BOLD activity. Therefore, as shown in Fig 4, the ME task in this study induced slightly slower but also the deepest and longest alpha power suppression. The MI task performance is sort of in between and facilitates both the instruction-related and the execution-related brain activity, as suggested in a study by Miller et al. [31], in which brain areas involved in both kinds of brain activity were reported to be associated with MI task performance. As a result, the average ERD response time was comparable to that for the ME task, but had only half the amplitude and a much shorter duration in terms of the alpha power suppression than that for the ME task.

However, one could argue that the difference in the arrival times for the MO and ME conditions might simply be caused by that the ME condition has deeper alpha power suppression than the MO condition. As a result, reaching the half-way point from the baseline to the maximum suppression might take longer in the ME condition as compared to the MO condition. Given that the suppression level (depth) of the ERD response in the MI condition is similar to that of the ERD response in the MO condition (Fig 5), if the arrival time difference is totally caused by the deeper ERD process in the ME condition, we should be able to see a significant difference in the arrival time between the MI and ME conditions as well.

### Brief ERD windows for the MI and MO conditions

One of the purposes for this study was to show how reliable the ERDs induced by MI and MO tasks are in order to determine their suitability for use as control mechanisms in BCI applications. Given the preprocessing step of ICA, it is possible to remove the contamination from irrelevant brain or non-brain processes mixed into the channel EEG data during signal acquisition [38, 39]. The results of this study may thus make it possible to determine the best scenarios (i.e., with minimum artifact contamination) for eliciting motor-induced ERD responses for reference in devising motor-imagery-based BCI applications. Given very brief and unstable ERD responses to the MO task (duration of ~300 ms and amplitude around 2.5 dB), it might be very difficult, if not impossible, to use the ERD responses induced by movement observation task for a BCI-based application.

On the other hand, although the ERD responses to the MI condition seemed to be slightly more pronounced than those to the MO condition, the average duration of the ERD responses and the amplitude of alpha power suppression might still be very difficult for online real-time detection (Fig 4). Such a difficulty may explain at least in part why real-world applications based on motor-imagery BCI-controlled systems are still very limited in the literature [40]. In order to better detect MI-induced ERD features for online real-time BCI applications, it may be essential to have better EEG signal quality as well as a higher sampling rate in the EEG acquisition system to provide better and more extensive EEG data for more effective online feature detection of MI-induced ERD. At the same time, a sensitive method for finding MI-induced ERD is also desirable for real-time control in a motor-imagery-based BCI system. Finally, an online evaluation mechanism for assessing the success rate on a trial-by-trial basis may also be needed if a MI-based BCI system is used for a BCI-controlled rehabilitation training system. By using such a mechanism, one could potentially determine if the limited effectiveness of a given type of BCI-controlled rehabilitation training is really caused by the poor performance in online MI feature detection or by the rehabilitation training protocol based on the MI-based BCI.

## Conclusion

By applying ICA to the combined EEG data derived from three types of motor tasks, this study demonstrates that all three of those different types of motor tasks, namely, motor execution, motor imagery, and movement observation tasks, can successfully induce alpha suppression in shared motor-related brain circuits. However, the motor-related alpha suppression induced by each type of task differs in terms of both depth and duration of suppression of the alpha EEG power. Because ICA plays an important role here in cleaning the EEG data by separating the motor-related independent EEG process from the artifactual components and other non-motor-related brain processes, the results of this work could provide the most optimistic estimation of how motor imagery- or motor observation-induced ERD can be used for BCI applications. Our results suggest that, at best, only 3–4 dB of suppression from the baseline, with around 500 ms duration starting at 500 ms after an execution cue is delivered, can be used as an EEG MI feature for a BCI application. If further taking into account the contamination in the EEG channel data in a regular BCI setup, more efficient methods of feature extraction and higher quality EEG data may be essential to success.

## References

[1] Jeannerod M (2001) Neural simulation of action: a unifying mechanism for motor cognition, Neuroimage 82: 1533–1539. 10.1006/nimg.2001.0832 11373140

[2] Munzart J, Lorey B, Zentgraf K (2009) Cognitive motor processes: The role of motor imagery in the study of motor representations, Brain Research Reviews 60: 306–326. 10.1016/j.brainresrev.2008.12.024 19167426

[3] Sharma N, Pomeroy VM, Baron J-C (2006) Motor imagery: a backdoor to the motor system after stroke? Stroke 37: 1941–1952. 16741183 10.1161/01.STR.0000226902.43357.fc

[4] Boe S, Gionfriddo A, Kraeutner S, Tremblay A, Little G, Bardouille T (2014) Laterality of brain activity during motor imagery is modulated by the provision of source level neurofeedback, Neuroimage 101: 159–167. 10.1016/j.neuroimage.2014.06.066 24999037

[5] Braun SM, Beurskens AJ, Borm PJ, Schack T, Wade DT (2006) The effects of mental practice in stroke rehabilitation: A systematic review, Archives Physical Medicine and Rehabilitation 87: 842–852. 10.1016/j.apmr.2006.02.034 16731221

[6] Jackson PL, Lafleur MF, Malouin F, Richards CL, Doyon J (2003) Functional cerebral reorganization following motor sequence learning through mental practice with motor imagery, Neuroimage 20: 1171–1180. 14568486 10.1016/S1053-8119(03)00369-0

[7] Page SJ, Szaflarski JP, Eliassen JC, Pan H, Cramer SC (2009) Cortical plasticity following motor skill learning during mental practice in stroke, Neurorehabilitation and Neural Repair 23: 382–388. 10.1177/1545968308326427 19155350 PMC3258452

[8] Rodriguez-Bermudez G, Garcia-Lanecina PJ, Roca-Dorda J (2013) Efficient automatic selection and combination of EEG features in least squares classifiers for motor imagery brain-computer interfaces, International Journal of Neural Systems 23(4): 1350015 10.1142/S0129065713500159 23746288

[9] Pfurtscheller G, Neuer C (1997) Motor imagery activates primary sensorimotor area in humans, Neurosci Lett. 239: 65–68. 9469657 10.1016/s0304-3940(97)00889-6

[10] Pfurtscheller G, Neuer C (2001) Motor imagery and direct brain-computer communication, Proceedings of the IEEE 89: 1123–1134.

[11] Neuper C, Scherer R, Wriessnegger S, Pfurtscheller G (2009) Motor imagery and action observation: Modulation of sensorimotor brain rhythms during mental control of a brain-computer interface, Clinical Neurophysiology 120: 239–247. 10.1016/j.clinph.2008.11.015 19121977

[12] Babiloni C, Babiloni F, Carducci F, Cincotti F, Cocozza G, Del Percio C, et al (2002) Human cortical electroencephalography (EEG) rhythms during the observation of simple aimless movements: a high-resolution EEG study, Neuroimage 17: 559–572. 12377134

[13] Cochin S, Barthelemy C, Lejeune B, Roux S, Martineay J (1998) Perception of motion and EEG activity in human adults, Clinical Neurophysiology 107: 287–295. 10.1016/s0013-4694(98)00071-6 9872446

[14] Hari R, Forss N, Avikainen S, Kirveskari E, Salenius S, Rizzolatti G (1998) Activation of human primary motor cortex during action observation: a neuromagnetic study, Proceeding National Academic Science USA. 95: 15061–15065. 10.1073/pnas.95.25.15061 PMC24575 9844015

[15] Muthukumaraswamy SD, Johnson BW, McNair NA (2004) Mu rhythm modulation during observation of an object-directed grasp, Brain Research 19: 195–201. 15019715 10.1016/j.cogbrainres.2003.12.001

[16] Obermen LM, McCleery JP, Ramachandran VS, Pineda JA (2007) EEG evidence for mirror neuron activity during the observation of human and robot actions: toward an analysis of the human qualities of interaction robots, Neurocomputing 70: 2194–2203.

[17] Pfsutscheller G, Scherer R, Leeb R, Keinrath C, Neuper C, Lee F, et al (2007) Viewing moving objects in virtual reality can change the dynamics of sensorimotor EEG rhythms, Presence Teleoperators and Virtual Environments 16: 111–118.

[18] Ertelt D, Small S, Solodkin A, Dettmers C, McNamara A, Binkofski F, et al (2007) Action observation has a positive impact on rehabilitation of motor deficits after stroke, Neuroimage 36: T164–T173. 17499164 10.1016/j.neuroimage.2007.03.043

[19] Szameitat J, Shen S, Conforto A, Sterr A (2012) Cortical activation during executed, imagined, observed, and passive wrist movements in healthy volunteers and stroke patients, Neuroimage 62: 266–280. 10.1016/j.neuroimage.2012.05.009 22584231

[20] Makeig S, Bell AJ, Jung T-P, Sejnowski TJ (1996) Independent component analysis of electroencephalographic data, Advanced Neural Information Processing Systems 145–151.

[21] Chou WC, Duann JR, She HC, Huang LY, Jung TP (2015) Explore the functional connectivity between brain regions during a chemistry working memory task, PLOS One 10: e0129019 10.1371/journal.pone.0129019 26039885 PMC4454549

[22] Onton J, Westerfield M, Twonsend J, Makeig S (2006) Imaging human EEG dynamics using independent component analysis, Neuroscience Biobehavioral Reviews 30: 808–822. 16904745 10.1016/j.neubiorev.2006.06.007

[23] Delorme A, Makeig S (2004) EEGLAB: an open source toolbox for analysis of single-trial EEG dynamics including independent component analysis, Journal of Neuroscience Methods 134: 9–21. 15102499 10.1016/j.jneumeth.2003.10.009

[24] Oostenveld R, Praamstra P (2001) The five percent electrode system for high-resolution EEG and ERP measurements, Clinical Neurophysiology 112: 713–719. 11275545 10.1016/s1388-2457(00)00527-7

[25] Delorme A, Palmer J, Onton J, Oostenveld R, Makeig S (2012) Independent EEG sources are dipolar, PLoS One: 10.1371/journal.pone.0030135 PMC3280242 22355308

[26] Leocani L, Toro C, Zhuang P, Gerloff C, Hallett M (2001) Event-related deschronization in reaction time paradigms: a comparison with event-related potentials and corticospinal excitability, Clinical Neurophysiology 112: 923–930. 11336910 10.1016/s1388-2457(01)00530-2

[27] Babiloni C, Del Percio C, Vecchio F, Sebastiano F, Di Gennaro G, Quarato PP, et al (2016) Alpha, beta and gamma electrocorticographic rhythms in somatosensory, motor, premotor and prefrontal cortical areas differ in movement execution and observation in humans, Clinical Neurophysiology 127: 641–654. 10.1016/j.clinph.2015.04.068 26038115

[28] Wang Y, Chen H, Gong Q, Shen S, Gao Q (2010) Analysis of functional networks involved in motor execution and motor imagery using combined hierarchical clustering analysis and independent component analysis, Magnetic Resonance Imaging 28: 653–660. 10.1016/j.mri.2010.02.008 20378292

[29] Hanakawa T, Dimyan MA, Hallett M (2008) Motor planning, imagery, and execution in the distributed motor network: A time-course study with functional MRI, Cerebral Cortex 18: 2775–2788. 10.1093/cercor/bhn036 18359777 PMC2583155

[30] Yuan H, Liu T, Szarkowski R, Rios C, Ashe J, He B (2010) Negative covariation between task-related responses in alpha/beta-band activity and BOLD in human sensorimotor cortex: An EEG and fMRI study of motor imagery and movements, Neuroimage 49: 2596–2606. 10.1016/j.neuroimage.2009.10.028 19850134 PMC2818527

[31] Miller KJ, Schalk G, Fetz EE, den Nijs M, Ogemann JG, Rao RP (2010) Cortical activity during motor execution, motor imagery and imagery-based online feedback, Proceeding National. Academic Science USA. 107: 4030–4035. 10.1073/pnas.0913697107 PMC2840149 20160084

[32] Mukamel R, Ekstrom AD, Kaplan J, Iacoboni M, Fried I (2010) Single-neuron responses in humans during execution and observation of actions, Current Biology 20: 750–756. 10.1016/j.cub.2010.02.045 20381353 PMC2904852

[33] Gerardin E, Sirigu A, Lehericy S, Poline JB, Gaymard B, Marsault C, et al (2000) Partially overlapping neural networks for real and imagined hand movement, Cerebral Cortex 10: 1093–1104. 11053230 10.1093/cercor/10.11.1093

[34] Zich C, Debener S, Kranczioch C, Bleichner MG, Gutberlet I, De Vos M (2015) Real-time EEG feedback during simultaneous EEG-fMRI identifies the cortical signature of motor imagery, Neuroimage 114: 438–447. 10.1016/j.neuroimage.2015.04.020 25887263

[35] Keysers C (2009) Mirror neurons, Current Biology 19: R971–R973. 10.1016/j.cub.2009.08.026 19922849

[36] Keysers C, Gazzola V (2010) Social neuroscience: Mirror neurons recorded in humans, Current Biology 20: R353–E354. 10.1016/j.cub.2010.03.013 21749952

[37] Gastaut HT, Bert J (1954) EEG changes during cinematographic presentation, Electroencephalography and Clinical Neurophysiology 6: 433–444. 13200415 10.1016/0013-4694(54)90058-9

[38] Jung TP, Makeig S, Humphries C, Lee TW, McKeown MC, Iragui V, et al (2000) Removing electroencephalographic artifacts by blind source separation, Psychophysiology 37: 163–178. 10731767

[39] Makeig S, Debener S, Onton J, Delorme A (2004) Mining event-related brain dynamics, Trends in Cognitive Science 8:204–210. 10.1016/j.tics.2004.03.008 15120678

[40] Kubler A, Holz E, Kaufmann T, Zockler C (2013) A user centered approach for bringing BCI controlled applications to end-users Recent Progress and Future Prospects, Fazel-Recai R. (Ed.), ISBN:978-953-51-1134-4, InTech.

